# Development of vaccines for SARS-CoV-2

**DOI:** 10.12688/f1000research.25998.1

**Published:** 2020-08-17

**Authors:** Wern Hann Ng, Xiang Liu, Suresh Mahalingam

**Affiliations:** 1 Emerging Viruses, Inflammation and Therapeutics Group, Menzies Health Institute Queensland, Griffith University (Gold Coast Campus), Queensland, Australia

**Keywords:** COVID-19, Vaccines, SARS-CoV-2

## Abstract

COVID-19 emerged in late 2019 and has rapidly spread through many countries globally. The causative SARS-CoV-2 virus was not known until recently, and there is little or no natural immunity in human populations. There is an urgent need for vaccines and drugs to combat this new pandemic. In just a few months, huge efforts and resources by government, academia, and industry have been thrown into the race to develop a vaccine. This brief review summarizes and discusses the array of technologies being applied to vaccine development, highlighting the strengths and weaknesses of the various approaches.

## Introduction

The first quarter of 2020 has been plagued by the emergence of a novel coronavirus disease, COVID-19 or severe acute respiratory syndrome coronavirus 2 (SARS-CoV-2). SARS-CoV-2 was first identified in Wuhan, China, and has since spread globally at an alarming rate, with over 200 countries and territories being infected ^1, 2^. In March 2020, SARS-CoV-2 was declared a global pandemic by the World Health Organization (WHO) ^3^, and as of 18 July 2020, there are over 14 million confirmed cases with over 594,000 deaths worldwide. These figures are expected to rise ( https://www.who.int/emergencies/diseases/novel-coronavirus-2019/situation-reports) ^2^. With the emergence of SARS-CoV-2, there are currently over 2,400 listed studies on the National Institute of Health database ( https://clinicaltrials.gov/ct2/results?cond=COVID-19) aimed at identifying a solution to this pandemic. The development of a much-needed vaccine against SARS-CoV-2 is of utmost importance. To date, there are more than 100 vaccine candidates being developed by industry and academic institutions using a wide range of technologies including live attenuated, viral vectored, DNA/RNA-based, protein-based, and inactivated vaccines ^4– 6^. At the time of writing, 19 vaccines are recorded as being in clinical trials ( Table 1) ^4– 7^. This article highlights various technologies employed in vaccine development for COVID-19, potential hurdles, and recent advances.

**Table 1.** Vaccine platforms and their current status.

Technology employed	Developer	Type of vaccine candidate	Current stage in clinical evaluation
**Non-** **replicating** **viral vector**	University of Oxford/AstraZeneca	ChAdOx1-S	Phase III
	CanSino Biologics Inc and Beijing Institute of Biotechnology	Adenovirus type 5 vector	Phase II
	Shenzhen Geno-Immune Medical Institute	Lentivirus modification	
	Gamaleya Research Institute	Adeno-based	Phase I
	GeoVax/BravoVax	Modified Vaccinia Ankara (MVA)-encoded virus-like particle (VLP)	Pre-clinical
	Stabilitech Biopharma Ltd	Oral Ad5 S	
	Janssen Pharmaceutical Companies	Ad26 (alone or witd MVA boost)	
	Altimmune	Adenovirus-based NasoVAX expressing SARS-CoV-2 Spike protein	
	Greffex	Ad5 S (GREVAX™ platform)	
	Vaxart	Oral vaccine platform	
	DZIF – German Center for Infection Research	MVA-S encoded	
	IDIBAPS-Hospital Clinic, Spain	MVA-S	
	Greffex	Ad5 S (GREVAX™ platform)	
	Centro Nacional de Biotecnología (CNB-CSIC), Spain	MVA expressing structural proteins	
	Reitdera/LEUKOCARE/Univercells	Replication defective Simian adenovirus (gRAd) encoding SARS-CoV-2 S	
	Valo tderapeutics Ltd	Adenovirus-based + HLA-matched peptides	
	National Center for Genetic Engineering and Biotechnology (BIOTEC)/GPO, Thailand	Inactivated flu-based SARS-CoV2 vaccine + adjuvant	
	University of Manitoba	Dendritic cell-based vaccine	
	University of Georgia/University of Iowa	Parainfluenza virus 5 (PIV5)-based vaccine expressing tde Spike protein	
	Bharat Biotech/Thomas Jefferson University	Recombinant deactivated rabies virus containing S1	
	Massachusetts Eye and Ear/Massachusetts General Hospital/AveXis	Adeno-associated virus vector (AAVCOVID)	
	ImmunityBio, Inc. and NantKwest, Inc.	[E1-, E2b-, E3-] hAd5-COVID19-Spike/nucleocapsid	
	National Research Centre, Egypt	Influenza A H1N1 vector	
**RNA**	Moderna/NIAID	Lipid nanoparticle (LNP)-encapsulated mRNA	Phase II
	BioNTech/Fosun Pharma/Pfizer	Three LNP-mRNAs	Phase I/II
	CureVac	mRNA	Phase I
	People's Liberation Army (PLA) Academy of Military Sciences/ Walvax Biotechnology		
	Imperial College London	LNP-nCoVsaRNA	
	University of Tokyo/Daiichi-Sankyo	LNP-encapsulated mRNA	Pre-clinical
	Fudan University/Shanghai JiaoTong University/RNACure Biopharma	LNP-encapsulated mRNA cocktail encoding VLP/receptor-binding domain (RBD)	
	China CDC/Tongji University/Stermina	mRNA	
	Arcturus Therapeutics/Duke-NUS Medical School		
	FBRI SRC VB VECTOR, Rospotrebnadzor, Koltsovo		
	Greenlight Biosciences		
	IDIBAPS-Hospital Clinic, Spain		
	BIOCAD	Liposome-encapsulated mRNA	
	Centro Nacional de Biotecnología (CNB-CSIC), Spain	Replicating defective SARS-CoV-2-derived RNAs	
	Translate Bio/Sanofi Pasteur	LNP-mRNA	
	CanSino Biologics/Precision NanoSystems		
	Chula Vaccine Research Center/University of Pennsylvania		
	RNAimmune, Inc.	Several mRNA candidates	
	eTheRNA	mRNA in an intranasal delivery system	
**DNA**	INOVIO Pharmaceuticals	DNA plasmid vaccine with electroporation device	Phase I/II
	Cadila Healthcare Limited	DNA plasmid vaccine	Phase I/II (not yet recruiting)
	Genexine Consortium	DNA vaccine (GX-19)	Phase I
	Takis Biotech/Applied DNA Sciences/EvviVax	DNA	Pre-clinical
	BioNet-Asia	DNA vaccine	
	Entos Pharmaceuticals		
	Mediphage Bioceuticals/University of Waterloo	msDNA vaccine	
	Zydus Cadila	DNA plasmid vaccine	
	Karolinska Institute/Cobra Biologics (OPENCORONA Project)	DNA with electroporation	
	Chula Vaccine Research Center		
	Osaka University/AnGes/Takara Bio	DNA plasmid vaccine	
	Immunomic Therapeutics, Inc./EpiVax, Inc./PharmaJet, Inc.	Plasmid DNA, needle-free delivery	
	Symvivo	bacTRL-Spike	
	Scancell/University of Nottingham/ Nottingham Trent University	DNA plasmid vaccine RBD and N	
	National Research Centre, Egypt	DNA plasmid vaccine S, S1, S2, RBD, and N	
**Inactivated**	Sinovac	Formaldehyde-inactivated + alum	Phase III (not yet recruiting)
	Beijing Institute of Biological Products/Sinopharm	Inactivated	Phase I/II
	Wuhan Institute of Biological Products/Sinopharm		
	Institute of Medical Biology, Chinese Academy of Medical Sciences		Phase I
	Osaka University/BIKEN/NIBIOHN	Unknown	Pre-clinical
	Sinovac/Dynavax	Inactivated + CpG 1018	
	Valneva/Dynavax		
	National Research Centre, Egypt	Inactivated whole virus	
	Beijing Minhai Biotechnology Co., Ltd.	Inactivated	
	Research Institute for Biological Safety Problems, Rep of Kazakhstan		
**Live** **attenuated** **virus**	Codagenix/Serum Institute of India	Deoptimized live attenuated vaccines	Pre-clinical
	Indian Immunologicals Limited/Griffith University	Codon de-optimization of live attenuated vaccine	
	UMC Utrecht/Radboud University	Recombinant BCG (rBCG) technology	
**Protein** **subunit**	Novavax	Full-length recombinant SARS CoV-2 glycoprotein nanoparticle vaccine adjuvanted with Matrix (M)	Phase I/II
	Clover Biopharmaceuticals Inc./GSK/Dynavax	Native like trimeric subunit Spike protein vaccine	Phase I
	Anhui Zhifei Longcom Biopharmaceutical/Institute of Microbiology, Chinese Academy of Sciences	Adjuvanted recombinant protein (RBD-dimer)	
	Vaxine Pty Ltd/Medytox	Recombinant Spike protein with Advax™ adjuvant	
	ExpreS2ion Biotechnologies	Drosophila S2 insect cell expression system VLPs	Pre-clinical
	Osaka University/BIKEN/National Institutes of Biomedical Innovation, Japan	VLP recombinant protein + adjuvant	
	Chulalongkorn University/GPO, Thailand	RBD protein fused with Fc of immunoglobulin G + adjuvant	
	AdaptVac (PREVENT-nCoV consortium)	Capsid-like particle	
	Helix Biogen Consult, Ogbomoso, and Trinity Immonoefficient Laboratory, Ogbomoso, Oyo State, Nigeria	Subunit	
	WRAIR/USAMRIID	S protein	
	AJ Vaccines		
	EpiVax/University of Georgia		
	National Institute of Infectious Diseases, Japan	S protein + adjuvant	
	Sanofi Pasteur/GSK	S protein (baculovirus production)	
	University of Virginia	S subunit intranasal liposomal formulation with GLA/3M052 adjuvants	
	ImmunoPrecise/LiteVax BV	Spike-based (epitope screening)	
	Vaxil Bio	Peptide	
	Flow Pharma Inc		
	IMV Inc	Peptide antigens formulated in lipid nanoparticle formulation	
	Generex/EpiVax	Ii-Key peptide	
	EpiVax	Protein subunit EPV-CoV-19	
	National Research Centre, Egypt	Protein subunit S, N, M and S1 protein	
	Heat Biologics/University of Miami	gp-96 backbone	
	University of Queensland/GSK/Dynavax	Molecular clamp stabilized Spike protein	
	Baylor College of Medicine	S1 or RBD protein	
	iBio/CC-Pharming	Subunit protein, plant produced	
	VIDO-InterVac, University of Saskatchewan	Adjuvanted microsphere peptide	
	LakePharma, Inc.	Nanoparticle vaccine	
	Baiya Phytopharm/Chula Vaccine Research Center	Plant-based subunit (RBD-Fc + adjuvant)	
	Biological E Ltd	Adjuvanted protein subunit (RBD)	
	University of Saskatchewan	Adjuvanted microsphere peptide	
	University of Pittsburgh	Microneedle arrays S1 subunit	
	Saint-Petersburg Scientific Research Institute of Vaccines and Serums	Recombinant protein, nanoparticles (based on S-protein and other epitopes)	
	Innovax/Xiamen University/GSK	COVID-19 XWG-03 truncated S (Spike) proteins	
	OncoGen	Synthetic long peptide vaccine candidate for S and M proteins	
	MIGAL Galilee Research Institute	Oral *Escherichia coli*-based protein expression system of S and N proteins	
	Lomonosov Moscow State University	Structurally modified spherical particles of the tobacco mosaic virus (TMV)	
	University of Alberta	Spike-based	
	AnyGo Technology	Recombinant S1-Fc fusion protein	
	Yisheng Biopharma	Recombinant protein	
	Vabiotech	Recombinant S protein in IC-BEVS	
	Applied Biotechnology Institute, Inc.	Orally delivered, heat-stable subunit	
	Medigen Vaccine Biologics Corporation/NIAID/Dynavax	S-2P protein + CpG 1018	
	National University of San Martin and CONICET, Argentina	Protein subunit	
	MOGAM Institute for Biomedical Research, GC Pharma		
	Axon Neuroscience SE	Peptides derived from Spike protein	
	Intravacc/EpiVax	Outer membrane vesicle (OMV) subunit	
		OMV peptide	
	Neovii/Tel Aviv University	RBD-based	
	Kentucky Bioprocessing, Inc		
	Quadram Institute	OMV-based vaccine	
	BiOMViS Srl/University of Trento		
	FBRI SRC VB VECTOR, Rospotrebnadzor, Koltsovo	Peptide vaccine	
		Subunit vaccine	
**Virus-like particles**	Medicago Inc./Université Laval	Plant-derived VLP	Pre-clinical
	Saiba GmbH	VLP based on RBD displayed on VLPs	
	Navarrabiomed, Oncoimmunology group	VLPs, lentivirus and baculovirus vehicles	
	VBI Vaccines Inc.	Enveloped VLP (eVLP)	
	Mahidol University/The Government Pharmaceutical Organization (GPO)/Siriraj Hospital	VLP + adjuvant	
	IrsiCaixa AIDS Research Institute/IRTA-CReSA/Barcelona Supercomputing Centre/Grifols	S protein integrated in HIV VLPs	
	Imophoron Ltd and Bristol University Max Planck Centre	ADDomer™ multiepitope display	
	Doherty Institute	Unknown	
	OSIVAX	VLP	
	University of Sao Paulo	VLPs/whole virus	
	ARTES Biotechnology	eVLP	
**Replicating** **viral vector**	Shenzhen Geno-Immune Medical Institute	Minigenes engineered based on multiple viral genes, using an efficient lentiviral vector system (NHP/TYF) to express viral proteins and immune modulatory genes	Phase I
	Cadila Healthcare Limited	Measles vector	Pre-clinical
	Institute Pasteur/Themis/University of Pittsburg Center for Vaccine Research		
	FBRI SRC VB VECTOR, Rospotrebnadzor, Koltsovo		
	DZIF – German Center for Infection Research/CanVirex AG	Measles virus (S, N targets)	
	Tonix Pharmaceuticals/Southern Research	Horsepox vector expressing S protein	
	University of Hong Kong	Influenza vector expressing RBD	
	IAVI/Merck	Replication-competent vesicular stomatitis virus (VSV) chimeric virus technology (VSVΔG) delivering the SARS-CoV-2 Spike (S) glycoprotein	
	BIOCAD and IEM	Live viral vectored vaccine based on attenuated influenza virus backbone (intranasal)	
	Lancaster University, UK	Avian paramyxovirus vector (APMV)	
	FBRI SRC VB VECTOR, Rospotrebnadzor, Koltsovo	Recombinant vaccine based on influenza A virus for the prevention of COVID-19 (intranasal) VSV vector	
	University of Western Ontario	VSV-S	
	Israel Institute for Biological Research/Weizmann Institute of Science		
	Fundação Oswaldo Cruz and Instituto Butantan	Attenuated influenza expressing an antigenic portion of the Spike protein	
	Intravacc/Wageningen Bioveterinary Research/Utrecht University	Newcastle disease virus vector (NDV-SARS-CoV-2/Spike)	
	UW–Madison/FluGen/Bharat Biotech	M2-deficient single replication (M2SR) influenza vector	
**Unknown**	Tulane University	Unknown	Pre-clinical

The table was adapted from ^5, 6, 21^.

## DNA/RNA-based platforms

DNA- and RNA-based platforms present the greatest potential for speed of production since culturing and fermentation are not required for their synthesis. The potential of this approach is showcased by Moderna, whose vaccine candidate advanced to clinical testing just 2 months after sequence identification ^7^. There are many other advantages associated with DNA-based vaccines. Notably, they are renowned for their safety profile since the vectors employed are non-replicating and encode and express only the target antigen. The vectors therefore are unable to revert to a disease-causing form, which is a risk with viral vectors. Another key advantage with DNA-based vaccination is the absence of vector-specific immunity, which allows these products to be utilized in prime and boost regimens with multiple products intended for the same patient ^8^. RNA-based vaccines are also a promising alternative owing to their potential for low-cost manufacture and good safety profile in animal studies ^9– 11^. However, both DNA- and RNA-based vaccines have their own set of challenges. Both vaccines could suffer from the drawback of having low immunogenicity, as DNA vaccines could potentially integrate into the human genome, while there are concerns about the stability of RNA vaccines ^12^. As there are currently no approved DNA or RNA vaccines for medical use in humans ^12, 13^, the question about low immunogenicity has not yet been resolved ( Table 2). The lack of currently approved DNA or RNA vaccines provides considerable regulatory uncertainty, and their progression through the regulatory process will almost certainly take significantly longer than for more conventional vaccine technologies. Currently, there is one vaccine in phase II clinical trials, three in phase I/II clinical trials, and four in phase I clinical trials for SARS-CoV-2 based on the DNA/RNA platform. Inovio Pharmaceuticals is developing a SARS-CoV-2 vaccine, INO-4800, using a DNA-based platform, with phase I clinical trials being undertaken in conjunction with pre-clinical studies in order to hasten development ^14^. The International Vaccine Institute (IVI) has announced that the Coalition for Epidemic Preparedness Innovations (CEPI) has awarded $6.9 million of funding to INOVIO to work with IVI and the Korea National Institute of Health (KNIH) for a phase I/II clinical trial of INO-4800 in South Korea. The trial will be conducted in parallel with INOVIO’s phase I study, which is currently ongoing with 40 healthy adults receiving the vaccine candidate and will eventually be expanded to older adults ^15^. An RNA vaccine candidate, mRNA-1273, is being developed by Moderna in collaboration with NIAID. The Biomedical Advanced Research and Development Authority (BARDA), a division of the Office of the Assistant Secretary for Preparedness and Response (ASPR) within the US Department of Health and Human Services (HHS), has agreed to cooperate to hasten the development of mRNA-1273 by pledging up to $483 million in funding ^16^. Healthy adults aged 18 to 55 were recruited for phase I trials. Two injections of mRNA-1273 were given 28 days apart at a dose of 25 μg, 100 μg, or 250 μg. Dire adverse events were absent, and no trial halting rules were met, although one participant in the 25 μg group withdrew because of an unsolicited adverse event, transient urticaria, which was postulated to be linked to the first vaccination. Live virus neutralization capable of reducing SARS-CoV-2 infectivity by 80% or more was detected at day 43 post-vaccination in all participants. Of the three doses evaluated, the 100 μg dose was optimal based on its capacity to trigger high neutralization responses and Th1-skewed CD4 T cell responses. The reactogenicity profile at 100 μg was better than for the other two doses. The safety, reactogenicity, and immunogenicity of mRNA-1273 are currently being evaluated in phase II trials using two vaccinations given 28 days apart. A total of 600 participants, 300 aged 18–54 and 300 aged 55 and above, have been assigned to three groups: placebo, 50 μg of vaccine, or 100 μg of vaccine at both vaccinations ^17^. A phase III trial of mRNA-1273 is expected to begin in July with an estimated 30,000 participants. With the expected final dose of 100 μg, Moderna is confident to scale up manufacturing to approximately 500 million to 1 billion doses per year ^18^.

**Table 2.** Pros and cons of different vaccine formulations and examples of licensed vaccines.

Vaccine platforms	Pros	Cons	Examples of licensed vaccines targeted for humans
**RNA**	• Potential low-cost manufacturing • Ease of manufacturing • Good safety profile	• May have low immunogenicity due to instability • May require multiple doses	-
**DNA**	• Potential low-cost manufacturing • Ease of manufacturing • Good safety profile • Good stability • Does not induce anti-vector immunity	• Potential integration to human genome • Low immunogenicity	-
**Virus vectors** **(replicating/non-** **replicating viral vectors** **and virus-like particles)**	• High-efficiency gene transduction • High specific delivery of genes to target cells • Induction of robust immune responses • Increased cellular immunity	• Low titer production • May induce anti-vector immunity • Generation of replication- competent virus, which can induce tumorigenesis	• JYNNEOS (Smallpox/ Monkeypox) • ACAM2000 (Smallpox) • Adenovirus type 4 and type 7 vaccine, live, oral (febrile acute respiratory)
**Inactivated**	• Good safety profile • Can be used in immunocompromised patients	• Requires booster doses • Low production titer	• Poliovax (Polio) • Flucelvax Quadrivalent (Influenza) • Ixiaro (Japanese Encephalitis) • Imovax (Rabies)
**Live attenuated virus**	• High potency • Triggers long-lasting immunity • Low-cost manufacturing	• Possible regression to virulence strain • Limited use in immunocompromised patients	• ERVEBO (Ebola virus) • MMR II (Measles, Mumps, and Rubella) • BCG vaccine (Tuberculosis)
**Protein subunit**	• Can be used in immunocompromised patients • Good safety profile	• Low immunogenicity • Conjugation could lead to batch-wise variation	• PedvaxHIB ( *Haemophilus* *influenzae* type b) • Engerix-B (Hepatitis B) • Recombivax HB (Hepatitis B)

This table was adapted from ^8– 11, 20, 22– 28^.

## Virus vectors

Virus-based vectors are powerful tools for vaccination. Their effectiveness stems from their ability to infect cells, which allows them to be highly efficient, specific, and able to trigger robust immune responses. Despite their advantages, viral vectors have several disadvantages ( Table 2). For example, the use of vaccinia virus and adenovirus may lead to immunity against the vector, which will reduce the efficacy of the vaccine. The use of retrovirus and lentivirus, on the other hand, may lead to the risk of tumorigenesis in patients, which results from the integration of viral long terminal repeats into proto-oncogenes ^19^. Additionally, certain viral vectors such as adeno-associated virus may not be cost effective owing to their low titer production ^20^. Ultimately, viral vectors still present themselves as a valid choice for vaccine development against SARS-CoV-2, as illustrated by their successful use in the eradication of smallpox ^20^. Examples of such vaccines currently under development are ChAdOx1-S, Ad5-nCoV, aAPC, and LV-SMENP-DC. The latter two vaccines, developed by Shenzhen Geno-Immune Medical Institute, are currently in phase I (aAPC) and phase II (LV-SMENP-DC) clinical studies. The Ad5-nCoV vaccine pioneered by CanSino Biologics is the first SARS-CoV-2 vaccine to reach phase II clinical trials ^5^. Phase I clinical studies for Ad5-nCoV were conducted between March 16 and March 27. No serious adverse events within 28 days of vaccination were reported in vaccine recipients. Specific antibodies, including neutralizing antibodies, increased significantly at day 14 and peaked 28 days post-vaccination. Specific T-cell response peaked at day 14 post-vaccination ^29^. A phase II study is currently underway with 500 participants registered in three different groups: 250 participants will receive the vaccine, 125 participants will receive a lower vaccine dose, and 125 participants will receive placebo. The immune response will be tested at 0, 14, and 28 days and 6 months after vaccination ^30^. ChAdOx1-S, developed by the University of Oxford in partnership with AstraZeneca, is the first vaccine candidate to reach phase III clinical trials ^1, 5^. A study has reported that a single dose of the vaccine is able to elicit a strong immune response in rhesus macaques ^31^. Additionally, a trial study conducted in pigs demonstrated that ChAdOx is able to elicit a greater antibody response when given a booster shot, suggesting that a two-dose approach may give better protection in humans against SARS-CoV-2 ^32^. However, it should be noted that nose swabs and oropharynx and mediastinal lymph node testing revealed viral gRNA in both vaccinated and control animal groups at 3 and 5 days post-inoculation. Interestingly, viral genome was detected in cervical lymph node in the vaccinated group but not in the control group. Detection of virus in these tissues indicates the vaccine does not provide complete protection against SARS-CoV-2 infection. Viral RNA (gRNA and sgRNA) load in lung tissue was also shown to vary dramatically between individual animals but was determined to be significantly lower in the vaccinated group than in the control group. Another troubling result to note from this study is that despite observing neutralizing antibodies in vaccinated animals, the titers reported were extremely low ^31^. Generally, neutralizing antibodies elicited by effective vaccines can be diluted more than a thousand-fold and still maintain their effectiveness ^33^. However, the results reported by Oxford show that the serum could be diluted only 4- to 40-fold before losing its neutralizing activity ^31, 33^. ChAdOx1-S was well tolerated in humans, with a trial of over 320 vaccinated individuals showing no strong adverse effects ^34^. The phase III clinical trials of this vaccine will involve 8,000 individuals in the United Kingdom, 5,000 individuals in Brazil, and 2,000 individuals in South Africa ^35^. AstraZeneca has reached an agreement with Europe’s Inclusive Vaccines Alliance (IVA) to supply up to 400 million doses of ChAdOx1-S starting by the end of 2020 at no profit. The total manufacturing capacity of this vaccine currently stands at 2 billion doses ^34^.

## Inactivated vaccines

Inactivated vaccines have been successfully employed over the past 70 years and are widely used today ^36^. Inactivated vaccines are produced using bacteria or viruses by deactivating them with heat, chemicals, or radiation. These processes terminate the pathogen’s ability to replicate, leading to them being more stable and having higher safety profiles. These attributes allow for their use in immunocompromised individuals ^37^. However, the characteristics that contribute to their strengths are also weaknesses. As the pathogens are inactivated, these vaccines generally stimulate a much weaker immune response than live vaccines and require several doses for effective immunity to be established. The immune response to an inactivated vaccine is also typically humoral. Antibody titers against the targeted antigen will diminish with time, leading to the need for booster shots ^22, 36^ ( Table 2). Regardless, inactivated vaccines are effective agents that have prevented countless deaths due to various infections in humans, notably against wild poliovirus 2, which has not been detected since 1999 and was declared eradicated in September 2015 by WHO ^38^. There are currently nine inactivated vaccine candidates for SARS-CoV-2 listed by WHO. Of these, one vaccine candidate—PiCoVacc, developed by Sinovac—is listed as ready to commence phase III clinical trials and is in the midst of preparing to recruit participants. It is very promising to note that Sinovac Biotech has demonstrated protection by PiCoVacc against SARS-CoV-2 in monkeys. Monkeys were immunized three times with two different doses (3 or 6 μg per dose) of PiCoVacc at day 0, 7, and 14 before virus challenge at week 3. It was demonstrated that monkeys vaccinated with PiCoVacc produced anti-SARS-CoV-2 neutralizing antibody titers similar to those of recovered patients. This study also indicated that PiCoVacc is safe, as there was no infection enhancement or immunopathological exacerbation observed in vaccinated monkeys. PiCoVacc is currently in phase I human clinical trials as stated above. The company plans to initiate phase II and III clinical trials with PiCoVacc by the end of this year ^39^. There are two other inactivated vaccine candidates being developed by the Beijing Institute of Biological Products and the Wuhan Institute of Biological Products, respectively, both of which are currently in phase I/II clinical trials. Both institutes are collaborating with Sinopharm for the development of these vaccines.

## Live attenuated virus

Another promising approach in vaccine design is the use of live attenuated virus (LAV), developed by codon deoptimization. This technology has proven itself to be cost effective for large-scale manufacturing and has a smooth regulatory approval pathway, as it has demonstrated high efficacy and potency in both *in vitro* and *in vivo* experiments against different respiratory viruses in mice, human cells, and non-human primates, providing lasting immunity with just a single dose ^23– 25^. LAV have a simple production process using well-established Vero cells, which are currently the most widely accepted cell line by regulatory authorities for vaccine development, since they have been in use for vaccine manufacturing for nearly 40 years. LAVs are also proven to grow and infect microcarrier beads in large-scale fermenters up to 6,000 L and in serum free media with zero loss in production output ^40^. Existing manufacturing infrastructure can be easily utilized because of its cost effectiveness for large-scale manufacturing ^41^. LAV has certain drawbacks, notably the existing possibility of reversion to virulence, which has strong safety implications, especially in immunocompromised patients ^26^ ( Table 2). Nonetheless, codon deoptimization technology in vaccine design has promise against SARS-CoV-2. It is a tried and tested new engineering technology, and designs such as introducing point mutations have proved that the drawbacks can be overcome with relative ease ^42^. One example of its recent use is in a vaccine against Ebola virus, where an estimated efficacy of 97.5% was recorded from a preliminary analysis of 90,000 individuals who were exposed for 10 days or more to Ebola virus after vaccination ^43^. Codon deoptimization vaccine candidates have virtually no risk of reversion to virulence because of the large number of substitutions that are made in the coding sequence. This is a crucial safety feature of vaccines developed using codon deoptimization. Vaccines developed against SARS-CoV-2 using LAV technology are all currently in pre-clinical development. For example, Indian Immunologicals Limited is currently working together with Griffith University to develop a vaccine using codon deoptimization as a strategy against SARS-CoV-2. The vaccine candidate is expected to provide a long-lasting immunity against SARS-CoV-2 following a single vaccination and is also expected to provide cross-protection against other coronaviruses such as MERS and SARS-CoV-1 ^44^.

## Protein subunit

Protein subunit vaccines are a popular choice for the design of a SARS-CoV-2 vaccine owing to their strong safety profile, which is particularly advantageous for immunocompromised patients. They are less likely to cause complications in vaccinated individuals, as the antigenic components employed in protein subunit vaccines are purified and do not involve the use of infectious viruses ^45^. However, as with other vaccine platforms, protein subunit vaccines come with their own set of challenges, with the most prominent one being their lack of efficacy. A subunit vaccine presents an antigen to the immune system without the involvement of viral particles using a specific, isolated protein of the pathogen. In the likely scenario where the isolated proteins are denatured, they will become associated with other antibodies different from what was initially targeted, leading to the lack of efficacy ^46^ ( Table 2). Nonetheless, novel designs and delivery can overcome this limitation and demonstrate enhanced efficacy, e.g. against Zika virus in animal studies and against malaria in *in vivo* studies ^47, 48^. The success of protein subunit vaccines is highlighted by the hepatitis B vaccine, and we have now controlled these diseases to the point of virtual elimination ^49, 50^. Currently, there are many vaccines under pre-clinical studies using this platform. For example, Danish company Expression Biotechnologies has announced that it was awarded an EU horizon 2020 grant amounting to €2.7 million for the development of a SARS-CoV-2 vaccine candidate. The company aims to conduct phase I/IIa clinical trials within 12 months after preliminary studies ^51^. One other notable example of a vaccine candidate designed on the basis of this platform is developed by Novavax, which is currently in phase I/II clinical trials. There are three other protein subunit vaccine candidates currently in phase I clinical trials ^5^.

## Outlook

SARS-CoV-2 has given rise to an unprecedented economic, health, and societal challenge globally. The key to tackle this pandemic is the development of safe and effective vaccines. Monumental energy has been poured into vaccine R&D; however, there are many challenges that need to be overcome before a successful marketable vaccine becomes available. Over 100 vaccine candidates are in pre-clinical trials, with 19 in clinical trials and many others in early development ^5, 6^. Vaccine developers are turning a blind eye to industrial benchmarks for traditional vaccine development, as speed is considered a major priority in developing a successful vaccine against SARS-CoV-2. For example, clinical trials are being conducted simultaneously instead of sequentially using adaptive designs that are optimized for speed. Researchers, manufacturers, funders, and governing parties are targeting vaccines to be available by 2021, impelling proposals to move forward with reduced numbers of study participants and cut short safety follow-up in clinical studies. A successful COVID-19 vaccine will be administered on a global scale; thus, its safety profile must be solid. Therefore, approaches for generating a COVID-19 vaccine must not compromise safety aspects and will require careful evaluation of effectiveness and safety at each step to avoid major setbacks. There are also unresolved obstacles in ensuring global access to a developed COVID-19 vaccine. For example, during the early days of the HIV and H1N1 outbreaks, even after the successful development of a vaccine, it was difficult to achieve global distribution. Similarly, for COVID-19, a vaccine approved for marketing does not necessarily lead to easy accessibility. Manufacturing capability must be enhanced so that the production of vaccine doses can be magnified while ensuring vaccine affordability for the global community. Coordinated efforts among vaccine developers, funders, and manufacturers are essential to ensure that successful vaccine candidates can be manufactured in adequate numbers and distributed evenly across the globe.

Another important issue is whether COVID-19 vaccines will sensitize humans to a phenomenon known as antibody-dependent enhancement (ADE). Although antibodies are generally protective, rare cases have been documented in which pathogen-specific antibodies enhance disease—a phenomenon known as ADE. ADE is most prominently associated with dengue virus ^52^. ADE has also been observed for both MERS and SARS-CoV-1 despite evidence pointing towards the fact that coronavirus diseases in humans lack the clinical, epidemiological, biological, or pathological attributes of ADE exemplified by the dengue viruses. Therefore, ADE warrants full consideration in the safety profiling of potential vaccine candidates against SARS-CoV-2 to avoid this phenomenon being observed, as SARS-CoV-2 shares a high sequence identity with SARS-CoV-1 ^53, 54^.

Safety aspects of the COVID-19 vaccines under development are of the utmost importance. Thorough clinical trials need to be carried out prior to marketing of the vaccine. Recent reports in India on the possibility of launching a vaccine in record time without allowing sufficient time for clinical trials are disturbing. Although COVID-19 vaccine development is of the utmost global importance, it is crucial that we do not cut corners and that vaccines are approved only after having passed through rigorous clinical trials.

## Concluding remarks

There are an extraordinary number of COVID-19 vaccines currently under development around the world. Highly optimistic timeframes of 12–18 months to get to the distribution of approved vaccines are being touted. Vaccines typically take many years or even decades to develop, e.g. the smallpox vaccine took 25 years to develop for human use, the HPV vaccine 15 years, the rotavirus vaccine 15 years, and the influenza vaccine 28 years. The COVID-19 vaccine initiatives aspire to have vaccines ready for distribution by 2021. This is a truly remarkable and unprecedented timeframe for vaccine development. The majority of vaccines won’t progress beyond pre-clinical or early clinical testing, but, of course, we need only one effective COVID-19 vaccine to halt this pandemic. Fewer than 10% of drugs or vaccines that enter clinical trials ever progress to become available for human use. For example, 30% fail at phase I trials. Of those that progress to phase II, 69% fail and then a further 42% fail at phase III. Of those products that successfully pass through phase III trials, a further 15% do not gain regulatory approval. Because of the drastically shorter time being allotted for the pre-clinical development of COVID-19 vaccine candidates, it is conceivable that a higher proportion of these candidates may fail during clinical testing. In addition to vaccine development, a number of resource-intensive activities will need to be undertaken in parallel, particularly the construction of manufacturing facilities capable of producing at a global scale. We are hopeful the unmatched resources being directed into the international vaccine R&D effort will lead to a successful vaccine capable of stopping this new pandemic.
